# Development and validation of the BASE-66 inventory for comprehensive academic stress measurement

**DOI:** 10.1371/journal.pone.0343308

**Published:** 2026-03-11

**Authors:** Juan Luis Castillo-Navarrete, Claudio Bustos, Alejandra Guzmán-Castillo, Lorena Muñoz-Reveco, Walter Zavala

**Affiliations:** 1 Departamento de Tecnología Médica, Facultad de Medicina, Universidad de Concepción, Concepción, Biobío, Chile; 2 Programa de Doctorado en Salud Mental, Facultad de Medicina, Universidad de Concepción, Concepción, Biobío, Chile; 3 Programa de Neurociencia, Psiquiatría y Salud Mental, NEPSAM, Universidad de Concepción, Concepción, Biobío, Chile; 4 Departamento de Psicología, Facultad de Ciencias Sociales, Universidad de Concepción, Concepción, Biobío, Chile; 5 Departamento de Ciencias Básicas y Morfología, Facultad de Medicina, Universidad Católica de la Santísima Concepción, Concepción, Biobío, Chile; 6 Departamento de Ciencias Clínicas y Preclínicas, Facultad de Medicina, Universidad Católica de la Santísima Concepción, Concepción, Biobío, Chile; 7 Carrera de Fonoaudiología, Facultad de Ciencias de Salud y Ciencias Sociales, Universidad de las Américas, Concepción, Biobío, Chile; Prague University of Economics and Business: Vysoka Skola Ekonomicka v Praze, CZECHIA

## Abstract

Academic stress (AS) is a multidimensional process expressed through emotional, cognitive, physiological, and behavioral responses to academic demands. The COVID-19 pandemic accelerated the digitalization of higher education and restricted in-person social interaction, increasing the salience of socioeconomic constraints, home-based learning conditions, and interactional disruptions. These changes exposed measurement gaps in widely used AS instruments, particularly in their coverage of contextual stressors and in the psychometric consistency of coping-related content. To address this gap, we developed and provided initial psychometric validation for the BASE-66 (Broad Academic Stress Evaluation), an inventory designed to assess AS through an integrated framework that includes both academic and non-academic sources of strain. We used an instrumental psychometric design implemented in three phases: item development, main administration, and post-test evaluation. BASE-66 was adapted from the SISCO-II-AS framework through qualitative focus groups conducted during emergency remote teaching, followed by pilot testing and iterative item refinement. The main administration included 501 university students (71.7% women; mean age 22.19 years, SD=3.13) recruited from three universities in Concepción, Chile. Factor structure was examined with exploratory factor analyses (EFA) based on polychoric correlations using WLSMV estimation in a stratified split sample (EFA n=254; CFA n=247). Confirmatory factor analysis tested the full retained solution. Reliability was assessed with Cronbach’s alpha and McDonald’s omega in the full sample, and temporal stability was evaluated via test–retest correlations in a follow-up subsample (n=85). Stressor items supported a five-factor structure: Academic Workload Stressors, Academic Performance Stressors, Stressors associated with General Social Interaction, Socioeconomic Stressors, and Stressors associated with Classroom Interaction. Reaction items were best represented by a bifactor model with a dominant General Reaction factor and three specific domains capturing residual variance: Emotional–Cognitive Reactions, Physical Exhaustion Reactions, and Social Conflict Reactions. Coping items suggested two factors (Restorative Coping and Distraction–Reappraisal Coping), but their loadings and reliability were comparatively weaker, supporting a cautious interpretation. The confirmatory model showed acceptable fit (χ²/df=1.28; RMSEA=0.037; SRMR=0.077; CFI=0.93). Internal consistency was adequate to excellent across stressor and reaction domains, and test–retest correlations indicated solid stability for several scales, with lower stability for social and classroom interaction stressors consistent with higher contextual sensitivity. Women scored higher on multiple stressor and reaction domains, whereas classroom interaction stressors and several coping indices did not show gender differences. In conclusion, BASE-66 provides a context-sensitive instrument for comprehensive AS assessment in Chilean university students, capturing academic, socioeconomic, and interactional stressor domains alongside differentiated reaction profiles. Future work should evaluate measurement invariance, strengthen coping measurement, and incorporate longitudinal and multi-method validation designs.

## Introduction

Stress is a neuroendocrine, immunological, and behavioral response that organisms experience in response to demands imposed by their environment. Exposure to a stressor may activate adaptive processes that elicit acute or chronic responses [1–3]. Academic stress (AS) refers to the tension experienced in educational contexts. The onset of AS may occur as early as primary school and generally intensifies as students progress along their educational trajectory, reaching higher levels during the university years [4,5]. Higher education represents a particularly demanding stage, largely due to the volume and complexity of academic workload. University students are frequently exposed to periods of heightened academic demand that require substantial adjustment efforts. When these demands exceed available resources, AS may be associated with burnout, reduced engagement with studies, and difficulties in coping with academic challenges [6].

The term academic stress is often used in a broad or informal manner, despite the fact that its meaning and implications are not always clearly defined. This lack of conceptual precision is compounded by the wide range of terms employed to refer to stress in educational settings, including student stress, university stress, burnout, school stress, and exam stress.

In the university context, the assessment of stress has traditionally relied on instruments that provide relatively general and weakly contextualized measures. Some tools evaluate stressful situations associated with academic, family, and economic domains, such as the Student-Life Stress Inventory [7], the Undergraduate Sources of Stress Questionnaire [8], the Academic Expectation Stress Inventory [9] and the College Student Stress Scale [10]. Other instruments focus more specifically on the stressful potential of academic conditions, including the Academic Stressors Scale of the Academic Stressor Questionnaire (ECEA) [11–13].

In 2007, Arturo Barraza Macías proposed a theoretical model offering a broader, process-based account of academic stress. This model conceptualized academic stress as an adaptive systemic psychological process, integrating the appraisal of stressful situations, psychophysiological responses, and coping strategies. The process was described as unfolding across three moments: confronting demands appraised as stressors, a systemic imbalance expressed through symptoms, and the deployment of coping actions aimed at restoring balance [14]. Within this framework, three core components were identified: stressor stimuli, symptoms indicative of imbalance, and coping strategies.

Based on this model, Barraza developed in Mexico a self-report psychometric instrument, the SISCO Academic Stress Inventory (SISCO-AS), structured around three dimensions assessing stressors, symptomatology, and coping, as well as an overall score [15]. Although the SISCO-AS has been widely used and its psychometric properties have been examined [16–18], a reformulation of the instrument, the SISCO-II Academic Stress Inventory (SISCO-II-AS), was reported in 2020 for use in college student populations. This revised version retained the stressors and coping subscales and refined the symptomatology domain, referred to as total reaction, by distinguishing two factors: physical and psychological reactions, and social behavioral reactions [19].

The COVID-19 emergency led to the mandatory implementation of Emergency Remote Teaching (ERT), profoundly altering educational practices in higher education [20,21]. This abrupt transition substantially restricted social interactions with family members, peers, and the broader academic community, while simultaneously intensifying academic demands within the domestic environment. As a result, the boundaries between everyday life and academic activities became increasingly blurred.

This emerging socio-technological context highlighted the need to reassess the dimensions traditionally considered in the measurement of AS. Neither the SISCO-AS nor the SISCO-II-AS explicitly capture for several of these contemporary challenges, underscoring the importance of updating their conceptual and measurement frameworks. In addition, empirical studies have identified challenges in how coping is operationalized in both instruments, suggesting the need for further refinement [16,17,19]. Within this context, the development of a new instrument is justified to address relevant facets of AS that were not previously considered.

AS evaluation should consider both intra- and extra-university factors [22]. Recent evidence suggests that these interactions have become more salient following the COVID-19 pandemic, shaping students’ perceptions and responses to AS [23,24]. Moreover, the interplay between academic and non-academic stressors can have a sustained impact on student well-being in current higher education contexts. [25,26]. Notwithstanding their significance, comprehensive instruments capable of addressing these dimensions simultaneously remain scarce, highlighting an important measurement gap.

AS, which is ubiquitous among students, is closely linked to stressors within the educational environment. Various academic factors can induce this stress, including workload, evaluation demands, and organizational conditions [17,19]. Recent studies indicate that, in the postpandemic context, these academic stressors interact more intensely with personal and non-academic factors, amplifying their impact on students’ psychological well-being and academic functioning [23,24,26,27].

AS is influenced by students’ ability to navigate academic demands, which requires a range of competencies such as study strategies, organizational skills, effective communication, problem-solving, and time management. These resources are unevenly distributed among students, shaping a multifaceted framework for coping with AS [28–32]. Students who possess these competencies tend to display more effective coping responses, characterized by higher motivation and stronger organizational capacity, which are key for managing academic demands.

External personal resources, particularly non-financial ones, can also influence AS. Resources such as emotional support, supportive peer relationships, and available free time, play an important role. When students’ individual needs in these domains are not adequately met, academic stress may be further intensified.

Students’ emotional, cognitive, and physical needs play a central role in how they cope with academic demands. These needs influence learning styles, study preferences, work pace, and the balance between academic and personal life [33]. The interaction between individual needs and available resources can give rise to distinct academic stress profiles. While some students require calm and structured environments, others depend more heavily on social interaction and emotional support. For instance, students with high emotional needs but limited social support may experience increased levels of AS. Likewise, insufficient personal resources may overwhelm students when academic demands exceed their coping capacity [32,34].

Social safety theory posits that experiences of social safety or threat influence health and behavior, offering a useful framework for understanding AS responses. According to this perspective, the brain and immune system are biologically oriented toward maintaining safety by fostering social bonding and enabling anticipatory responses to potential threats [35,36]. During the COVID-19 pandemic, social disconnection emerged as an additional stressor for students [37] , with potential consequences for health, behavior, and increased levels of AS. Reduced social cohesion may have further intensified stress through pathways that involve immune functioning [38]. Variability in AS responses can influence students’ well-being and academic performance. While some students demonstrate resilience through effective coping strategies, others may experience heightened stress and greater difficulty managing academic demands.

Understanding the diversity of AS responses is essential for providing appropriate support and designing effective intervention strategies. These responses can be broadly organized into emotional, cognitive, and physiological dimensions. Emotional responses encompass experiences such as anxiety, fear, frustration, sadness, anger, and worry [39] . Cognitive responses relate to students’ appraisal of academic demands and may manifest as negative thoughts, critical self-evaluations, and concerns about academic performance. Physiological responses refer to bodily changes associated with stress, including sleep disturbances, muscle tension, headaches, gastrointestinal discomfort, and alterations in appetite [6].

The concept of AS underscores the relevance of adopting a multidimensional approach to its assessment and management, allowing a more comprehensive understanding of the phenomenon and supporting the development of more effective targeted interventions. Evaluating the risks associated with stressful situations requires considering how perceived stress affects health outcomes. This perspective provides a basis for designing interventions that are both more effective and better tailored to students’ needs [6].

The literature indicates that stress is experienced and managed differently by men and women as a result of biological, psychological, and sociocultural factors [40, 41,42,43]. These differences highlight the importance of considering both sex and gender in the assessment and management of AS, a need that becomes even more relevant given the increasing participation of women in higher education worldwide [44]. Furthermore, evidence suggests that the severity of perceived stress is a stronger predictor of mental and physical health outcomes than exposure to stressors alone [45]. This association appears to be particularly relevant in women, who report poorer sleep quality and a greater burden of physical and psychological complaints under conditions of high perceived stress, underscoring the central role of individual stress perception.

Instead of drawing an a priori boundary**,** the development of BASE-66 prioritized psychometric robustness by evaluating candidate domains empirically**,** with a focus on identifying coherent and interpretable structures across the instrument’s content coverage. Accordingly, the scale development examined stressor and response domains and explored the contribution of coping-related content within the same psychometric framework.

The aim of this study was to evaluate the psychometric properties of a scale designed to measure AS related stressors, responses, and coping strategies. The specific objectives were: (i) to develop an instrument that integrates academic and non-academic factors; (ii) to examine its factorial structure using exploratory and confirmatory factor analyses (EFA and CFA); and (iii) to assess its reliability through internal consistency and test-retest procedures.

The resulting instrument, named BASE-66 (Broad Academic Stress Evaluation), was developed to build on the SISCO-AS and SISCO-II-AS inventories by explicitly incorporating the interaction between academic and non-academic factors in the perception of stress and stress responses, while also evaluating coping-related content as part of the broader AS assessment. It is intended to provide a more comprehensive evaluation of the core components of AS in university students.

This study was guided by two main hypotheses aimed at evaluating the psychometric performance of BASE-66. The first hypothesis (H1) posits that the instrument will exhibit a coherent factorial structure aligned with its theoretical foundations, allowing the identification of key components previously described in the SISCO-II-AS framework. Specifically, it is expected that stressor-related items will be organized into distinct factors reflecting academic and broader social and contextual stressors. Regarding stress domains, it is anticipated that items will differentiate between physical and psychological reactions and social and behavioral reactions, with coping-related content examined as an additional domain within the same psychometric framework.

The second hypothesis (H2) addresses the reliability of the instrument, which will be examined through indicators of internal consistency and test-retest stability. It is expected that the overall scale and its subscales will demonstrate acceptable levels of internal consistency, as assessed using Cronbach’s alpha and Omega coefficients. Additionally, test-retest reliability is expected to reach adequate levels for each subscale, as estimated using Pearson’s correlation coefficient.

## Methodology

Designed to examine an instrument’s psychometric properties, this instrumental study (Ato et al., 2013) comprises three phases: item creation, main implementation, and post-test evaluation.

### Participants

A total of 501 students, with a mean age of 22.19 years (SD = 3.13), participated in the study. Of the total sample, 71.7% were female (n = 359). Participants came from three universities in Concepción, Chile, representing seven different faculties. Each participant provided online informed consent by completing an online informed consent form. The study was approved by the Scientific Ethical Committee of the Faculty of Medicine of the University of Concepción (CEC 30–2020) and by the Ethics, Bioethics and Biosafety Committee of the Vice-Rectory of Research and Development of the University of Concepción (CEBB 966–2021).

### Instrument

BASE-66, originally a 68-item questionnaire, begins with two introductory items. The first item measures the level of worry/nervousness using a 5-point scale. The second dichotomous item asks whether participants have experienced moments of worry/nervousness. Subsequently, the remaining 66 items were divided into two sections: Stressors and Reactions, with 33 items each. Additionally, a sociodemographic questionnaire was used to collect sociodemographic and educational information.

## Procedure

### Item formulation

The BASE-66 was initially developed as an adaptation of the SISCO-II-AS inventory [19]. This adaptation was designed to meet the demands of emergency remote teaching. As in the development of the SISCO-II-AS, the first step included focus groups with online university students. This process aimed to verify the tool’s relevance to emergency remote teaching and to identify stressors, reactions, and coping strategies that required the addition of new items.

We organized five focus groups: three at Universidad de Concepción (UdeC), one at Universidad Católica de la Santísima Concepción (UCSC), and one at Universidad de las América (UDLA). Each group, consisting of up to 11 students, was recruited throught organizations and directly by researchers. Initially, the SISCO-II-AS was applied to each group. Students were asked to identify stress-inducing scenarios, somatic reactions, and coping strategies within the emergency remote teaching context during COVID-19 that the SISCO-II-AS did not adequately capture.

### Focus group analysis

The team jointly reviewed the focus group responses, coding them to identify common categories. These included 19 stressor categories, 17 reaction categories, and 10 coping categories (see Supplementary Table 1). Depending on the complexity of each category, one or two items were added to the instrument. Consequently, a preliminary version of the BASE-66 comprising 98 items was tested, incorporating 36 additional stressor items to the original eight, 16 new reaction items to the existing 17, and 15 new coping items to the original six.

### Pilot testing and analysis

During the first half of November 2021, a pilot test was conducted with 112 participants using the preliminary version of BASE-66. The primary purpose of this pilot study was to provide initial evidence to inform a subsequent EFA with a larger sample, rather than to establish a definitive factor structure. A preliminary EFA was conducted for each dimension (stressors, reactions, and coping) to identify underlying subscales. To determine the number of subscales, Horn’s parallel analysis (PA) was used. Items with factor loadings above 0.40 were retained; additionally, some theoretically relevant items were preserved despite having loadings close to 0.40, given the reduced sample size and the expected instability of factor solutions under such conditions. Of the 44 stressor items in the preliminary version, 31 were retained as-is, 5 items were merged into 2 items, and 8 items were deleted, resulting in a 33-item stressor section. All 33 reaction items were retained as-is, yielding a 2-factor solution. For coping, the 21 items yielded a 7-factor solution; however, because four factors were composed of only two related items, they were transformed into four new items. Of the remaining 13 items, 12 were retained, with only one item deleted due to a low factor loading, resulting in a 16-item coping section. This pilot sample was excluded from subsequent analyses to strengthen the methodological rigor of the later EFA and CFA.

### Data

The data used are available at https://doi.org/10.48665/udec/O4F7YD

### Main Implementation and test-retest

The instrument’s deployment coincided with the end of the second academic semester of 2021 (November 2021 to February 2022). The instrument was administered through LimeSurvey, an online survey platform (LimeSurvey, 2023), hosted on our secure server. Out of 1015 system entries, we received 501 valid responses. To evaluate response stability in the post-pandemic context, we re-administered the instrument at the end of the first academic semester of 2022 (May to June 2022) to assess test-retest reliability of the scales. Of the initial 501 students, 85 participated in **the** second administration (17%). Coping-related content was retained for psychometric evaluation, and its structure and reliability were examined alongside the other domains.

### Analysis plan

We set a significance level of α = 0.05 and conducted all analyses using RStudio [46] and the lavaan package. To evaluate the factor structure of BASE-66, we performed separate Exploratory Factor Analyses (EFAs) for the stressor, reaction, and coping scales, followed by a Confirmatory Factor Analysis (CFA) to test the complete factorial solution obtained from the EFAs. The complete sample was divided in half using stratified random sampling, with strata formed by career and sex; the first half (n = 254) was used for the EFAs and the second half (n = 247) for the CFA.

Before estimating the EFAs, we examined whether the data were suitable for factor analysis using Bartlett’s test of sphericity and the Kaiser Meyer Olkin (KMO) test of sampling adequacy, both applied to the polychoric correlation matrix. Adequacy criteria included a significant result on Bartlett’s test and KMO values exceeding 0.8 [47,48]. To determine the number of factors, we primarily relied on Horn’s parallel analysis (PA), a robust criterion for factor retention [49,50]. Our implementation of PA involved 1,000 bootstrap samples, using non-parametric resampling from the matrix of polychoric correlations [49]. Following Lim and Jahng (2019) [51], we tested two additional solutions beyond the one indicated by the PA: one with an extra factor and one with one fewer.

Exploratory factor analyses were conducted using structural equation modeling in lavaan, employing the WLSMV estimator, which was also used in the CFA. Two types of models were tested in the EFAs for the stressor, reaction, and coping scales: simple-structure and bifactor models. In the simple-structure solution, estimated using oblimin rotation, we aimed for each item to show a salient loading of at least 0.40 on a single factor. In bifactor models, all items load on a general factor and on one or more group (residual) factors that account for variance not explained by the general factor. Items with loadings below 0.40 on all factors, with two or more loadings exceeding 0.40 on different factors, or showing high residual sums were evaluated for potential elimination based on their conceptual relevance. Based on these analyses, the stressor and coping scales were best represented by simple-structure solutions, whereas the reaction scale was better modeled using a bifactor approach.

The solution for all factors obtained in the EFAs was then tested using CFA on the second subsample. To evaluate model fit, we followed Hu and Bentler [52]: a nonsignificant χ² (p > 0.05), Root Mean Square Error of Approximation (RMSEA) below 0.06, Standardized Root Mean Square Residual (SRMR) below 0.08, and Comparative Fit Index (CFI) above 0.95 [52].

For each identified scale, we computed reliability using Cronbach’s α and total omega to estimate internal consistency in the whole sample [53]. Total omega, also known as composite reliability, was obtained using the omega function from the psych package in R [46]. Average variance extracted (AVE) was calculated using the AVE function from the semTools package. According to Fornell and Larcker [54], an AVE value below 0.50 indicates that variance attributable to measurement error exceeds the variance explained by the latent factor [54]. Discriminant validity was supported when correlations between a given factor and all other factors were lower than the square root of its AVE (SQRT AVE).

Bifactor model (stress reactions): In the analysis of stress reactions, both analytical and theoretical considerations supported the suitability of a bifactor model. This model posits a general factor underlying all items and two or more group factors representing specific theoretical dimensions, independent of the general factor. To derive a valid solution, a bifactor (S-1) model was fitted, featuring one general factor and four specific domains, of which only three were specified as group factors in the statistical model [55]. Model fit and essential unidimensionality were evaluated using indicators proposed by Rodriguez et al. [56,57]: Explained Common Variance (ECV > 0.70), Percent Uncontaminated Correlations (PUC > 0.70), general-factor hierarchical omega (ωh > 0.80), construct reliability (H > 0.70), and Average Factor Loadings (AFL < 0.30) for each group factor. In addition, subscale hierarchical omega was used to assess how reliably each specific dimension was measured after controlling for the general factor, with values above 0.30 considered substantial [58].

Test-Retest Sample: To evaluate test-retest reliability, we calculated Pearson’s correlation coefficient for all scales in the sample of 85 participants who completed the second administration.

## Results

The BASE-66 questionnaire begins with two introductory items. The first item indicated that 488 respondents (97.4%) reported experiencing worry or nervousness, while 2 (0.4%) reported not experiencing these feelings, and 11 (2.2%) did not respond. When rating their worry or nervousness on a 1–5 scale, the majority selected 5 (“always”) (40.5%, n = 192), followed by 4 (“almost always”) (40.5%, n = 192), and 3 (“sometimes”) (15.4%, n = 73), with fewer selecting 2 (“rarely”) (2.3%, n = 11) or 1 (“never”) (1.3%, n = 6).

Table 1 presents descriptive statistics for the BASE-66 stressor, reaction, and coping scales for the whole sample. Item distribution analyses predominantly revealed unimodal patterns, except for a bimodal distribution in reaction item 31. Stressor item means ranged from 1.89 for “Lack of resources to pay for internet connectivity” to 4.15 for “Teacher evaluations.” Reaction item means ranged from 1.75 for “Vertigo” to 4.28 for “Feelings of guilt for not fulfilling my academic activities according to my expectations.” Coping item means ranged from 1.93 to 3.52. Skewness and kurtosis values were generally within the −1 to +1 range, with some items showing more pronounced departures, particularly for skewness. Overall, departures from normality were more often characterized by negative kurtosis, suggesting flatter distributions, without a consistent pattern of strong skewness across the item set.

**Table 1:** Descriptive statistics for BASE-66 items (5 = 501).

Item	Description	M	SD	Skew	Kurtosis
					
1	Level of preoccupation or nervousness	4.17	0.87	−1.02	1.08
	**Stressors**				
Str 1	Overload of homework and academic work	4,11	0,88	−0,89	0,59
Str 2	The personality and temperament of the teachers	3,08	1,06	−0,03	−0,64
Str 3	Teachers’ evaluations (exams, essays, research papers, etc.)	4,15	0,85	−0,68	−0,36
Str 4	The type of work teachers ask you to do (map consultation, worksheets, essays, concept maps, etc.).	3,52	1,05	−0,41	−0,40
Str 5	Not understanding the topics covered in class.	3,56	1,23	−0,39	−0,93
Str 6	Class participation (answering questions, presentations, etc.).	3,28	1,29	−0,16	−1,09
Str 7	Limited time to do the work	3,83	1,12	−0,69	−0,36
Str 8	Group mates make faster progress on assignments and/or academic work.	3,16	1,35	−0,06	−1,17
Str 9	Existing career assessment regulations	2,77	1,28	0,23	−1,00
Str 10	Problems in managing and coordinating academic activities during the semester.	3,43	1,28	−0,31	−1,00
Str 11	Lack of support from my family and friends with my academic duties	2,43	1,38	0,58	−0,94
Str 12	Low participation in social activities	3,22	1,35	−0,17	−1,15
Str 13	Reduced social interactions with friends and/or classmates	3,35	1,33	−0,31	−1,06
Str 14	Not being satisfied and/or not meeting my expectations with my academic performance.	4,09	1,07	−0,99	0,06
Str 15	Possibility of failing one or more subjects	3,59	1,41	−0,51	−1,12
Str 16	Problems with Internet connection during my academic activities (stability, speed)	3,02	1,35	0,01	−1,17
Str 17	Problems with software and/or devices to connect to classes and do work (PC, telephone or other)	2,73	1,36	0,30	−1,11
Str 18	Problems working in a team with my classmates (scheduling difficulties, lack of cooperation, lack of communication).	3,08	1,31	−0,05	−1,13
Str 19	Physical and/or mental health problems	3,43	1,37	−0,39	−1,10
Str 20	Distractors while engaged in academic activities (pets, voices of family members, noises from neighbours, etc.)	3,62	1,24	−0,51	−0,83
Str 21	Lack of time to spend with family and/or friends due to the academic burden.	3,78	1,19	−0,73	−0,37
Str 22	Little university social life	3,64	1,24	−0,49	−0,83
Str 23	Missing or longing for university life	3,34	1,37	−0,31	−1,10
Str 24	Lack of help with health, social and/or economic problems.	3,03	1,35	0,01	−1,19
Str 25	Balancing other responsibilities (domestic and/or work) with my academic activities.	3,72	1,23	−0,65	−0,61
Str 26	The functioning of my university platform(s) (Blackboard, Canvas, Teams, Moodle, others).	2,45	1,21	0,47	−0,71
Str 27	Having to work to pay for my studies and/or household expenses.	1,97	1,42	1,13	−0,27
Str 28	Lack of resources to renew equipment for academic work.	2,32	1,39	0,62	−0,94
Str 29	Lack of resources to pay for internet connectivity.	1,89	1,21	1,24	0,46
Str 30	Lack of interaction between students in class	2,72	1,25	0,24	−0,91
Str 31	Lack of interaction between students and teachers in class	2,75	1,16	0,15	−0,77
Str 32	Lack of response to questions and/or activities proposed by the teacher.	2,88	1,22	0,21	−0,89
Str 33	Lack of adequate physical space to study	3,22	1,44	−0,19	−1,31
	**Reactions**				
Rea 1	Sleep disorders (insomnia or nightmares)	3,53	1,22	−0,48	−0,70
Rea 2	Chronic fatigue (permanent tiredness)	3,96	1,09	−0,91	0,06
Rea 3	Headaches or migraines	3,39	1,20	−0,25	−0,86
Rea 4	Digestion problems, abdominal pain or diarrhoea	3,24	1,30	−0,18	−1,08
Rea 5	Scratching, nail biting, rubbing, etc.	3,48	1,47	−0,51	−1,15
Rea 6	Drowsiness or increased need for sleep.	4,07	1,11	−1,02	0,07
Rea 7	Muscle aches and/or contractures	3,35	1,35	−0,36	−1,09
Rea 8	Skin reactions (rash, peeling, etc.)	2,48	1,51	0,52	−1,20
Rea 9	Restlessness (inability to relax and be calm)	3,99	1,11	−0,89	−0,07
Rea 10	Anxiety, distress or despair	4,07	1,07	−1,01	0,26
Rea 11	Increased or decreased food intake	3,86	1,26	−0,92	−0,22
Rea 12	Physical and mental exhaustion	4,27	0,89	−1,14	0,81
Rea 13	Hair loss	2,87	1,54	0,10	−1,49
Rea 14	Feelings of guilt for not fulfilling my academic activities according to my expectations	4,28	1,01	−1,38	1,20
Rea 15	Problems with attention, concentration and/or memory	4,03	1,06	−0,87	−0,12
Rea 16	Feelings of inadequacy and/or uselessness with regard to studies	3,90	1,25	−0,83	−0,46
Rea 17	Eye problems (eye strain, blurred vision, dryness, palpitations, irritation)	3,49	1,35	−0,46	−0,97
Rea 18	Involuntary body movements, tics (involuntary movement of legs, throbbing in eyes or faces)	3,05	1,45	−0,04	−1,35
Rea 19	Sweating	2,52	1,37	0,41	−1,07
Rea 20	Tremors	2,25	1,36	0,72	−0,74
Rea 21	Tendinitis	2,15	1,34	0,86	−0,53
Rea 22	Warmth in the ears	1,84	1,14	1,28	0,72
Rea 23	Vertigo	1,75	1,15	1,42	0,93
Rea 24	Tinnitus (recurrent or permanent ringing in the ear)	2,16	1,26	0,84	−0,39
Rea 25	Dry mouth	2,38	1,35	0,55	−0,97
Rea 26	Increased or decreased sexual desire	2,84	1,41	0,06	−1,26
Rea 27	Breathing disturbances (shortness of breath, suffocation).	2,73	1,44	0,24	−1,27
Rea 28	Feelings of depression and sadness (low mood)	3,83	1,20	−0,71	−0,54
Rea 29	Feelings of aggression or increased irritability	3,34	1,25	−0,32	−0,84
Rea 30	Sudden mood swings	3,23	1,31	−0,2	−1,02
Rea 31	Conflict or tendency to argue or dispute	2,77	1,38	0,24	−1,13
Rea 32	Isolation from others	3,44	1,30	−0,37	−1,01
Rea 33	Unwillingness to do your work as a student	4,10	1,04	−0,99	0,31
	Coping				
Cop 1	Consume junk food and/or drinks to energize and/or calm myself.	3,50	1,07	−0,32	−0,50
Cop 2	Consume healthy foods and/or drinks to energize and/or calm myself.	2,33	1,21	0,56	−0,71
Cop 3	Use substances (alcohol, tobacco, drugs, medications) to energize and/or calm myself.	2,71	1,26	0,26	−0,98
Cop 4	Seek calm by finding a quiet space.	2,85	1,28	0,11	−1,04
Cop 5	Organize my workspace and work resources.	3,48	1,19	−0,36	−0,75
Cop 6	Avoid or postpone academic tasks by doing other things.	3,38	1,25	−0,30	−0,91
Cop 7	Ask for spiritual or religious help.	2,18	1,50	0,87	−0,79
Cop 8	Sleep and/or rest before or after a stressful task.	2,61	1,23	0,34	−0,84
Cop 9	Be empathetic and compassionate toward myself; try to forgive myself.	3,25	1,31	−0,25	−1,05
Cop 10	Seek professional support.	1,93	1,39	1,24	0,08
Cop 11	Spend time with a loved one (family, pets, friends, etc.).	3,33	1,29	−0,28	−0,97
Cop 12	Engage in a pastime (physical activity, reading, watching series, social media, etc.).	3,52	1,23	−0,37	−0,88
Cop 13	Try to find something positive or beneficial in the stressful situation.	3,51	1,21	−0,42	−0,81
Cop 14	Talk it out and confide in someone (verbalize the situation that concerns me).	2,04	1,39	1,04	−0,35
Cop 15	Praise and reward myself.	2,80	1,35	0,17	−1,13
Cop 16	Develop an action plan to carry out my tasks.	2,91	1,37	0,02	−1,23

The polychoric correlation matrix was suitable for factor analysis (available at https://osf.io/c63ng/?view_only=4ff0b0a8b9704bdd8e1e5076b27dc992). Bartlett’s test was significant, χ²(3321) = 26050.89, p < 0.001, and the KMO was 0.82, indicating adequate sampling adequacy. Given the multidimensional structure of the instrument, separate factor analyses were conducted for the stressor, reaction, and coping scales.

### Exploratory factor analysis of stressor items.

Horn’s parallel analysis based on the polychoric correlation matrix suggested seven factors. Following Lim and Jahng [51], solutions between three and eight factors were examined [51]. An initial six-factor solution showed an overall simple structure; however, one factor was defined by only two items (Item 16, problems with Internet connection, and Item 17, problems with software and devices). Given the limited interpretability and stability of a two-item factor, these two items were removed, and a five-factor solution was retained. This solution showed adequate fit and was consistent with the study’s theoretical framework and the preliminary EFA.

Starting from the five-factor solution with 31 stressor items, seven items were removed through iterative refinement. Specifically, Items 9, 10, 26, 18, 19, and 2 were deleted due to factor loadings below 0.40 across successive solutions. The resulting 25-item solution was then evaluated. Item 20 (Distractors) showed a low loading (λ = 0.38) and a comparatively larger sum of residuals, and was therefore removed. The final stressor structure comprised five factors and 24 items, explaining 56.4% of the variance in item responses.

The stressor factor structure is presented in Table 2. Factor 1, Academic Workload Stressors, comprised Items 1, 21, 3, 4, and 7, with the highest loading observed for Item 1 (“Overload of homework and academic work”). Factor 2, Academic Performance Stressors, comprised Items 5, 8, 14, 15, and 6, with the highest loading observed for Item 5 (“Not understanding the topics covered in class”). Factor 3, Social Interaction Stressors, comprised Items 13, 12, 22, and 23, with the highest loading observed for Item 13 (“Reduced social interactions with friends and/or classmates”). Factor 4, Socioeconomic Stressors, comprised Items 27, 29, 28, 25, 24, 33, and 11, with the highest loading observed for Item 27 (“Having to work to pay for my studies and/or household expenses”). Finally, Factor 5, Classroom Interaction Stressors, comprised Items 31, 32, and 30, with the highest loading observed for Item 31 (“Lack of interaction between students and teachers in class”).

**Table 2:** Exploratory factor analysis of stressors.

Item	Description	Factor 1	Factor 2	Factor 3	Factor 4	Factor 5
	**Academic Workload Stressors**					
Str 1	Overload of homework and academic work	**0,72**	0,03	0,07	0,02	−0,07
Str 21	Lack of time to spend with family and/or friends due to the academic burden.	**0,63**	−0,15	0,20	0,15	−0,03
Str 3	Teachers’ evaluations (exams, essays, research papers, etc.)	**0,60**	0,32	−0,09	−0,08	0,07
Str 4	The type of work teachers ask you to do (map consultation, worksheets, essays, concept maps, etc.).	**0,58**	0,04	0,08	0,07	0,01
Str 7	Limited time to do the work	**0,51**	0,16	0,07	0,04	0,09
	**Academic Performance Stressors**					
Str 5	Not understanding the topics covered in class.	0,07	**0,71**	0,00	−0,03	0,10
Str 8	Group mates make faster progress on assignments and/or academic work.	−0,05	**0,70**	0,07	0,04	0,09
Str 14	Not being satisfied and/or not meeting my expectations with my academic performance.	0,10	**0,58**	0,20	0,11	−0,10
Str 15	Possibility of failing one or more subjects	0,22	**0,50**	−0,14	0,18	−0,15
Str 6	Class participation (answering questions, presentations, etc.).	0,02	**0,43**	0,02	0,07	0,01
	**Social interaction Stressors**					
Str 13	Reduced social interactions with friends and/or classmates	0,08	−0,02	**0,88**	−0,03	−0,01
Str 12	Low participation in social activities	−0,02	0,15	**0,83**	−0,01	0,01
Str 22	Little university social life	0,20	−0,14	**0,59**	0,07	0,19
Str 23	Missing or longing for university life	0,00	−0,01	**0,44**	0,00	0,32
	**Socioeconomic Stressors**					
Str 27	Having to work to pay for my studies and/or household expenses.	−0,17	0,05	−0,02	**0,87**	−0,09
Str 29	Lack of resources to pay for internet connectivity.	0,15	−0,04	−0,12	**0,85**	0,10
Str 28	Lack of resources to renew equipment for academic work.	0,07	−0,04	0,00	**0,77**	0,12
Str 25	Balancing other responsibilities (domestic and/or work) with my academic activities.	0,11	0,04	0,08	**0,60**	0,02
Str 24	Lack of help with health, social and/or economic problems.	0,10	0,13	0,26	**0,49**	0,03
Str 33	Lack of adequate physical space to study	−0,03	0,18	0,19	**0,46**	0,12
Str 11	Lack of support from my family and friends with my academic duties	−0,02	0,24	0,24	**0,45**	−0,06
	**Classroom Interaction Stressors**					
Str 31	Lack of interaction between students and teachers in class	−0,02	−0,01	0,00	0,00	**0,94**
Str 32	Lack of response to questions and/or activities proposed by the teacher.	0,09	0,12	−0,13	0,03	**0,74**
Str 30	Lack of interaction between students in class	−0,09	−0,02	0,20	0,09	**0,73**

### Reactions’ exploratory factor analysis.

Horn’s analysis based on the polychoric correlation matrix suggested four factors and two components, with one dominant factor emerging across the tested solutions. Solutions with one to five factors were examined. When simple-structure models were fitted for the two- to five-factor solutions, substantial cross-loadings were observed. Based on theoretical considerations, bifactor models were then evaluated, specifying one general factor and two to four group factors. The most coherent solution comprised one general factor and three group factors.

Inspection of residuals indicated that Item 7 (Muscle aches and/or contractures) showed comparatively high residual associations with several items and did not load meaningfully on any group factor; therefore, it was removed. The final reaction structure thus comprised one general factor and three group factors, based on 32 items.

Table 3 presents the bifactor solution for the reaction items. Loadings on the General Stress Reaction factor ranged from 0.31 to 0.86, indicating a broad continuum of stress response intensity. Group Factor 1, Emotional–Cognitive Reactions, included Items 14, 15, and 16, with the highest loading observed for Item 16 (“Feelings of inadequacy and/or uselessness with regard to studies”). Group Factor 2, Physical Exhaustion Reactions, included Items 18, 19, 20, 21, 22, 23, 24, and 25, with the highest loading observed for Item 20 (“Tremors”). Group Factor 3, Social Conflict Reactions, included Items 29, 30, and 31, with the highest loading observed for Item 31 (“Conflict or tendency to argue or dispute”). This solution explained 53.9% of the variance in item responses.

**Table 3:** Exploratory factor analysis of reactions.

Item	Description	General Factor	Specific factor 1	Specific factor 2	Specific factor 3
Rea 28	Feelings of depression and sadness (low mood)	**0,86**	0,08	−0,10	0,08
Rea 12	Physical and mental exhaustion	**0,85**	0,02	0,04	−0,24
Rea 10	Anxiety, distress or despair	**0,83**	0,01	−0,01	−0,12
Rea 2	Chronic fatigue (permanent tiredness)	**0,79**	−0,03	−0,11	−0,24
Rea 9	Restlessness (inability to relax and be calm)	**0,75**	−0,02	−0,01	−0,23
Rea 33	Unwillingness to do your work as a student	**0,75**	0,33	−0,12	0,02
Rea 1	Sleep disorders (insomnia or nightmares)	**0,70**	−0,05	0,12	−0,05
Rea 27	Breathing disturbances (shortness of breath, suffocation).	**0,66**	−0,06	0,28	−0,01
Rea 11	Increased or decreased food intake	**0,65**	0,18	0,19	0,19
Rea 32	Isolation from others	**0,62**	0,27	0,03	0,20
Rea 6	Drowsiness or increased need for sleep.	**0,61**	0,18	0,01	−0,13
Rea 3	Headaches or migraines	**0,56**	−0,02	0,22	−0,04
Rea 4	Digestion problems, abdominal pain or diarrhoea	**0,56**	0,06	0,16	0,01
Rea 8	Skin reactions (rash, peeling, etc.)	**0,56**	−0,10	0,25	−0,12
Rea 5	Scratching, nail biting, rubbing, etc.	**0,55**	−0,02	0,20	0,05
Rea 17	Eye problems (eye strain, blurred vision, dryness, palpitations, irritation)	**0,50**	0,09	0,32	−0,12
Rea 26	Increased or decreased sexual desire	**0,49**	0,02	0,17	0,29
Rea 13	Hair loss	**0,45**	0,13	0,39	−0,04
	**Emotional-Cognitive Reactions**				
Rea 16	Feelings of inadequacy and/or uselessness with regard to studies	**0,59**	**0,59**	−0,08	0,01
Rea 15	Problems with attention, concentration and/or memory	**0,63**	**0,52**	0,05	0,01
Rea 14	Feelings of guilt for not fulfilling my academic activities according to my expectations	**0,61**	**0,50**	−0,06	−0,06
	**Physical Exhaustion Reactions**				
Rea 20	Tremors	**0,53**	−0,02	**0,64**	0,00
Rea 22	Warmth in the ears	0,31	0,00	**0,59**	0,24
Rea 23	Vertigo	**0,55**	−0,01	**0,54**	−0,08
Rea 19	Sweating	0,39	−0,02	**0,52**	0,14
Rea 18	Involuntary body movements, tics (involuntary movement of legs, throbbing in eyes or faces)	**0,52**	−0,02	**0,43**	0,00
Rea 21	Tendinitis	0,35	−0,03	**0,43**	−0,02
Rea 24	Tinnitus (recurrent or permanent ringing in the ear)	0,35	0,07	**0,41**	0,10
Rea 25	Dry mouth	**0,48**	0,06	**0,41**	0,03
	**Social Conflict Reactions**				
Rea 31	Conflict or tendency to argue or dispute	**0,65**	0,01	−0,03	**0,54**
Rea 29	Feelings of aggression or increased irritability	**0,73**	−0,01	−0,08	**0,53**
Rea 30	Sudden mood swings	**0,79**	−0,06	−0,05	0,35

### Coping’s exploratory factor analysis.

Horn’s analysis based on the polychoric correlation matrix indicated four factors and three components. Solutions with two to five factors were tested, with one dominant factor emerging across all solutions. An initial two-factor solution appeared suitable, with most items forming a simple structure, but several items showed factor loadings below 0.40. Items 6, 7, 14, 15, 16, 10, 11, and 5 were iteratively deleted due to factor loadings below 0.40 across successive factor solutions. The final two-factor solution comprised 8 items and explained 43.8% of the variance in item responses.

Table 4 presents the factor structure of the coping items. The first factor, Restorative Coping, comprises Items 8, 2, 3, 4, and 9, reflecting activities oriented toward actively seeking tranquility and rest to manage stressful situations. The item with the highest loading was Item 8 (“Sleeping and/or resting before or after a stressful task”). The second factor, Distraction and Reappraisal Coping, comprises Items 12, 1, and 13, reflecting activities that do not aim at rest, but rather involve active distraction or cognitive reframing. The item with the highest loading was Item 12 (“Engaging in a hobby (physical activity, reading, watching series, social media, etc.)”).

**Table 4:** Exploratory factor analysis of coping.

Item	Description	Factor 1	Factor 2
	**Restorative coping**		
Cop 8	Sleep and/or rest before or after a stressful task	**0,79**	0,02
Cop 2	Consume healthy food and/or beverages to energize and/or calm myself.	**0,59**	−0,10
Cop 3	Use substances (alcohol, tobacco, drugs, medications) to energize and/or calm myself.	**0,57**	0,03
Cop 4	Seek calm by finding a quiet space.	**0,59**	−0,03
Cop 9	Be empathic and compassionate toward myself; try to forgive myself.	**0,42**	0,07
	Distraction and reappraisal coping		
Cop 12	Engage in a pastime (physical activity, reading, watching series, social media, etc.).	−0,01	**0,98**
Cop 1	Consume junk food and/or beverages to energize and/or calm myself.	−0,06	**0,55**
Cop 13	Try to find something positive or beneficial in the stressful situation.	0,31	**0,48**

### Confirmatory Factor Analysis.

**Fig 1:**
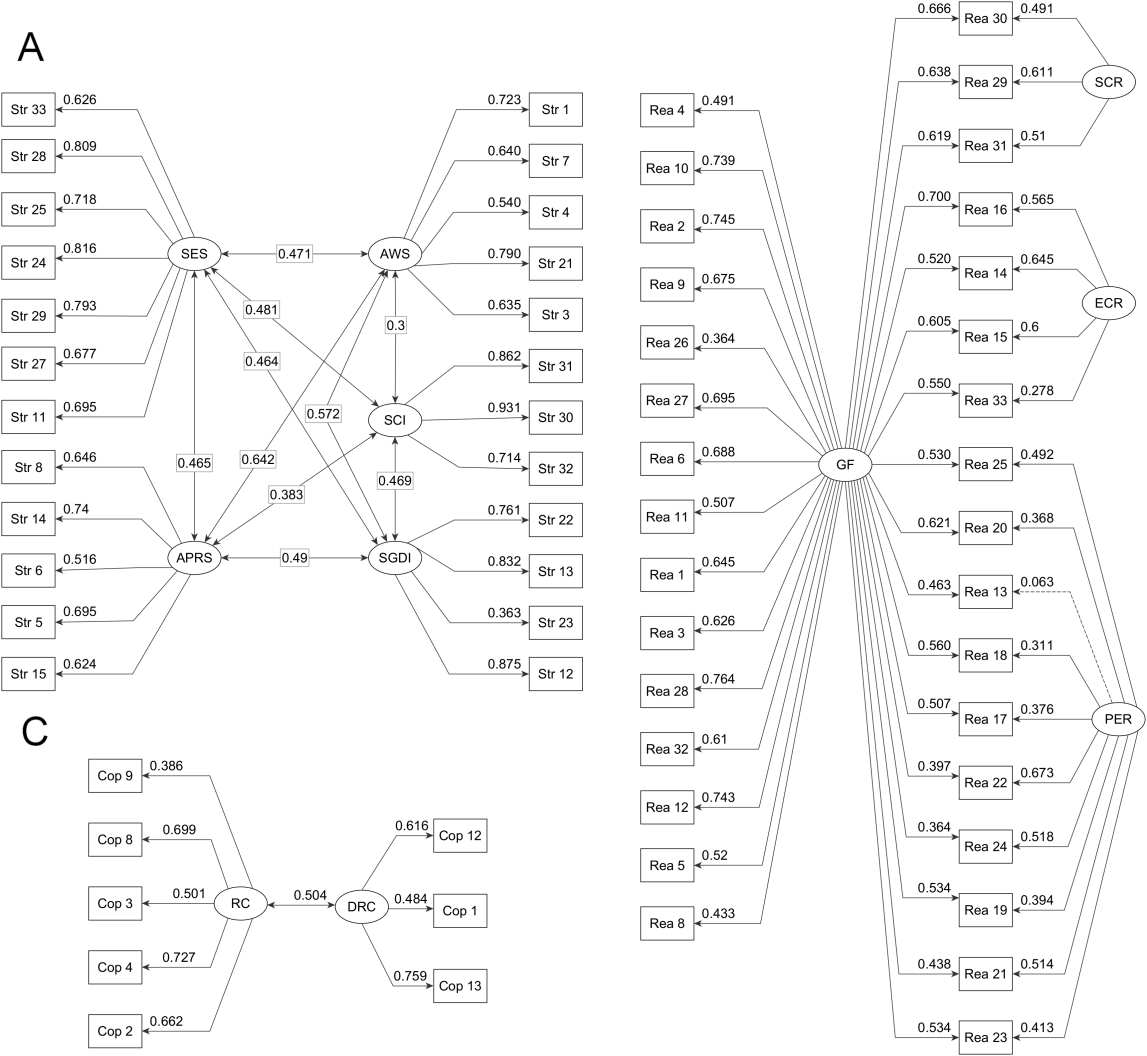
Standardized CFA solution for BASE-66 domains. Panel A: Stressors. Panel B: Reactions. Panel C: Coping. **AWS**: Academic Workload Stressors. **APRS**: Academic Performance Stressors. **SGDI**: Stressors associated with General Social Interaction. **SES**: Socioeconomic Stressors. **SCI**: Stressors associated with Classroom Interaction. **GF**: General Reaction. **ECR**: Emotional–Cognitive Reactions. **PER**: Physical Exhaustion Reactions. **SCR**: Social Conflict Reactions. **RC**: Restorative Coping. **DRC**: Distraction and Reappraisal Coping.

Confirmatory factor analysis (CFA) was performed on the second subsample (n = 247). The model derived from the exploratory analyses did not show exact fit to the data, χ²(1886) = 2415.97, p < .001, χ²/df = 1.28. Nevertheless, the model showed acceptable parsimonious fit, with RMSEA = 0.037, 95% CI [0.033, 0.041], p = 1.000, and SRMR = 0.077, both within recommended thresholds. The CFI value (0.93) remained below the conventional criterion of 0.95, indicating that a non-trivial proportion of residual variance was not captured by the specified structure. Overall, the combination of low error indices and theoretical coherence supports the adequacy of the proposed factorial solution. The standardized solution is presented in Fig 1.

Using bifactor-specific indices, the reaction scale showed clear dominance of a general factor, while specific factors retained meaningful but secondary relevance. The Explained Common Variance (ECV = 0.84) indicated that the general reaction factor accounted for 84% of the common variance, with the remaining 16% attributable to the specific domains of physical exhaustion and social conflict. Consistently, the Percent of Uncontaminated Correlations was high (PUC = 0.95), and the joint pattern of ECV and PUC values above 0.70 supports interpreting the reaction scale as essentially unidimensional. Nevertheless, several indicators suggested that the group factors contribute non-trivial reliable variance beyond the general factor. Hierarchical omega coefficients were substantial for Physical Exhaustion Reactions (ωh = 0.40) and Social Conflict Reactions (ωh = 0.35). The construct replicability index was excellent for the general factor (H = 0.96), acceptable for physical exhaustion (H = 0.67), and lower for social conflict (H = 0.58). Average factor loadings were above 0.30 for both physical exhaustion (AFL = 0.44) and social conflict (AFL = 0.57), supporting retention of the bifactor specification for interpretive purposes.

Table 5 presents the descriptive statistics for the stressor and reaction scales. Among stressors, *Academic Workload Stressors* showed the highest mean levels (M = 3.88), followed by *Academic Performance-Related Stress* (M = 3.53). In contrast, *Stressors associated with General Social Interaction* (M = 2.66) and *Stressors associated with Classroom Interaction* (M = 2.78) showed comparatively lower mean levels. Socioeconomic Stressors presented intermediate levels (M = 3.39). Regarding reactions, the *General Reaction* score was above the midpoint (M = 3.23), whereas *Physical Exhaustion Reactions* showed lower mean values (M = 2.45) and *Social Conflict Reactions* presented moderate levels (M = 3.12).

**Table 5:** Descriptors and reliability of stressors and stress reactions scales.

Scale (sub-scale)	Total		Women		Men		t test	p-value	ES(d)	Cronbach’s alpha	Total Omega	AVE	SQRT AVE
	M	SD	M	SD	M	SD							
Stressors													
Academic workload stressors	3.88	0.73	4	0.67	3.58	0.79	t(223.7)=5.59	<0.001	0.6	0.75	0.75	0.46	0.68
Academic Performance Stressors	3.53	0.89	3.63	0.86	3.3	0.93	t(241.4)=3.66	<0.001	0.38	0.74	0.74	0.42	0.65
Stressors associated with general social interaction	2.66	0.94	2.73	0.94	2.48	0.91	t(267.0)=2.77	0.006	0.27	0.82	0.82	0.58	0.76
Socioeconomic stressors	3.39	1.01	3.46	1	3.22	1.03	t(250.0)=2.32	0.021	0.23	0.76	0.78	0.54	0.73
Stressors associated with social interaction in the classroom	2.78	1.04	2.77	1.04	2.81	1.06	t(253.4)=0.34	0.738	0.03	0.83	0.83	0.70	0.84
Stressors Total	3.23	0.65	3.31	0.62	3.04	0.68	t(237.3)=4.00	<0.001	0.41	0.88	0.88	0.36	0.57
Reactions													
General Reaction	3.23	0.71	3.33	0.67	2.96	0.74	t(238.8)=5.28	<0.001	0.54	0.93	0.93 (0.88)	0.39	0.628
Emotional–Cognitive Reactions	4.08	0.88	4.16	0.83	3.87	0.98	t(223.8)=3.05	0.003	0.33	0.82	0.83 (0.28)	0.64	0.80
Physical Exhaustion Reactions	2.45	0.84	2.53	0.84	2.24	0.81	t(265.8)=3.46	<0.001	0.34	0.83	0.83 (0.37)	0.41	0.64
Social Conflict Reactions	3.12	1.15	3.23	1.13	2.82	1.15	t(255.2)=3.59	<0.001	0.36	0.85	0.85 (.32)	0.72	0.85
Coping													
Restorative Coping	2.75	0.85	2.73	0.86	2.81	0.82	t(269.9)=0.97	0.334	0.09	0.70	0.71	.36	0.60
Distraction and Reappraisal Coping.	3.51	0.89	3.61	0.87	3.26	0.89	t(254.5)=4.04	<0.001	0.4	0.63	0.66	0.44	0.67
Coping Total	3.04	0.71	3.06	0.71	2.98	0.72	t(255.7)=1.14	0.254	0.11	0.72	0.72	0.30	0.55

*M*: mean; *SD*: standard deviation; *p*-value: probability associated with the *t* statistic; *ES(d)*: Cohen’s *d*; *α*: Cronbach’s alpha; *ω*: McDonald’s omega; *ωH*: hierarchical omega; *AVE*: average variance extracted; *SQRT AVE*: square root of AVE.

The reliability analysis of the stressor scales showed adequate internal consistency across all stressor subscales. Academic Workload Stressors exhibited acceptable reliability (Cronbach’s α = 0.75; ω = 0.75), and Academic Performance-Related Stress showed comparable internal consistency (α = 0.74; ω = 0.74). Stressors associated with general social interaction and stressors associated with social interaction in the classroom demonstrated solid reliability, with Cronbach’s alpha values of 0.82 and 0.83, and total omega values of 0.82 and 0.83, respectively. Socio-economic stressors also showed adequate reliability (α = 0.76; ω = 0.78). At the total-score level, the stressors scale achieved high reliability (α = 0.88; ω = 0.88), supporting the use of a general stressor score.

Table 5 showed that Average Variance Extracted (AVE) values exceeded the recommended threshold of 0.50 for several dimensions, including Socio-economic stressors, stressors associated with general social interaction, stressors associated with social interaction in the classroom, and the reaction subscales Emotional–Cognitive Reactions and Social Conflict Reactions. This indicates that a substantial proportion of variance in the observed indicators is explained by their respective latent constructs, supporting the convergent validity of these domains.

Test–retest analyses supported the temporal stability of the BASE-66 stressor scales. Strong stability was observed for Academic Stressors (r = 0.74) and moderate stability for Socioeconomic Stressors (r = 0.52), with the total stressors score also showing solid stability over time (r = 0.64). In contrast, stressors related to general social interaction (r = 0.34) and classroom interaction (r = 0.48) showed lower stability, suggesting greater sensitivity to contextual changes across measurement occasions.

The reaction scales showed strong internal consistency. The General Reaction factor exhibited excellent reliability (Cronbach’s alpha = 0.93; total omega = 0.93), and the specific reaction subscales also demonstrated adequate reliability, including Physical Exhaustion Reactions (alpha = 0.80; omega = 0.80) and Social Conflict Reactions (alpha = 0.85; omega = 0.85). Test–retest correlations further supported the temporal stability of these scores for General Reaction (r = 0.74), Physical Exhaustion Reactions (r = 0.61), and Social Conflict Reactions (r = 0.62).

Significant gender differences were observed in several stressor and reaction measures, with women reporting higher mean scores where differences were present. Effect sizes ranged from negligible for Stressors associated with social interaction in the classroom (d = 0.03) to moderate for General Reaction (d = 0.54), indicating that the magnitude of gender differences varied across domains. Differences were significant for Academic Workload Stressors, Academic Performance-Related Stress, Stressors associated with general social interaction, Socio-economic Stressors, and the Total Stressors score, as well as for General Reaction, Emotional–Cognitive Reactions, Physical Exhaustion Reactions, and Social Conflict Reactions. No significant gender differences were observed for classroom interaction stressors, Restorative Coping, or the Coping Total score.

Our analysis of the single-item measure of self-perceived nervousness showed statistically significant correlations with all BASE-66 stressor and reaction scores reported in Table 6. The strongest association was observed with GF (r = 0.52, p < 0.01), followed by TS (r = 0.44, p < 0.01). Among stressor domains, nervousness showed moderate correlations with ASt (r = 0.41, p < 0.01), SGDI (r = 0.36, p < 0.01), and SES (r = 0.33, p < 0.01), whereas the association with SCI was small (r = 0.10, p < 0.05). Regarding reaction subscales, nervousness showed moderate correlations with SCR (r = 0.38, p < 0.01) and PER (r = 0.31, p < 0.01).

**Table 6:** Correlations between BASE-66 scales and subscales.

Scale/sub-scale	AWS	APRS	SGDI	SES	SCI	TS	GF	ECR	PER	SCR	RC	DCR	TC
Single item													
Self-perceived nervousness	0.48**	0.33**	0.32**	0.32 **	0.09	0.44 **	0.51 **	0.42 **	0.34 **	0.37 **	−0.13**	0.09	−0.05
Stressors													
AWS	--	0.43**	0.41**	0.4 **	0.22 **	0.68 **	0.54 **	0.45 **	0.41 **	0.36 **	−0.03	0.16 **	0.05
APRS		--	0.41**	0.3 **	0.22 **	0.69 **	0.47 **	0.6 **	0.3 **	0.3 **	−0.09 *	−0.01	−0.08
SGDI			--	0.38 **	0.39 **	0.81 **	0.54 **	0.37 **	0.45 **	0.38 **	−0.07	0.01	−0.05
SES				--	0.43 **	0.69 **	0.43 **	0.34 **	0.28 **	0.33 **	−0.02	0.07	0.01
SCI					--	0.59 **	0.29 **	0.18 **	0.26 **	0.23 **	0.12**	0.1 *	0.14**
TS						--	0.66 **	0.56 **	0.5 **	0.46 **	−0.05	0.07	0
Reactions													
GF							--	0.69 **	0.84 **	0.73 **	−0.05	0.12**	0.02
ECR								--	0.38 **	0.51 **	−0.16**	−0.01	−0.12**
PER									--	0.47 **	0.03	0.16**	0.10*
SCR										--	−0.03	0.06	0.01
Coping													
RC											--	0.33**	0.90**
DCR												--	0.71**

AWS: Academic Workload Stressors; APRS: Academic Performance Stressors; SGDI: Stressors associated with General Social Interaction; SES: Socioeconomic Stressors; SCI: Stressors associated with Classroom Interaction; TS: Total Stressors; GF: General Reaction; ECR: Emotional–Cognitive Reactions; PER: Physical Exhaustion Reactions; SCR: Social Conflict Reactions; RC: Restorative Coping; DRC: Distraction and Reappraisal Coping; TC: Total Coping; ** p < 0.01; * p < 0.05.

Stressor scale intercorrelations ranged from small to moderate, with the weakest association observed between Academic Stressors and Classroom Interaction Stressors (r = 0.27) and the strongest between Academic Stressors and Socioeconomic Stressors (r = 0.52). Regarding links between stressors and reactions, General Reaction showed its highest correlations with Academic Stressors (r = 0.59), Socioeconomic Stressors (r = 0.55), and Stressors associated with General Social Interaction (r = 0.50), whereas its association with Classroom Interaction Stressors was smaller (r = 0.29). Reaction subscales were strongly related to General Reaction (PER: r = 0.77; SCR: r = 0.73), and both showed moderate associations with Total Stressors (PER: r = 0.49; SCR: r = 0.46), supporting coherent convergence between stressor domains and the overall reaction construct.

In the discriminant validity analysis, we compared SQRT AVE values (Table 5) with inter-factor correlations. At the subscale level, most correlations were lower than the corresponding SQRT AVE values, supporting adequate discriminant validity across domains. This pattern weakened for aggregated scores. Specifically, the correlation between TS and GR (r = 0.67) exceeded the SQRT AVE for both TS (SQRT AVE = 0.57) and GR (SQRT AVE = 0.63), indicating substantial overlap when the instrument is interpreted at a general score level.

## Discussion

This study reports the initial validation of the BASE-66 (Broad Academic Stress Evaluation), a self-report inventory adapted from the SISCO-II-AS framework to assess AS-related stressors and reactions in Chilean university students while explicitly integrating academic and non-academic sources of strain that became particularly salient during emergency remote education. Grounded in Barraza-Macías’ systemic processual model of AS [14], and aligned with the SISCO-II-AS framework [59], BASE-66 was developed through a qualitative-informed revision and expansion process, based on focus groups conducted during the emergency remote education period, to broaden content coverage toward domains that the post-pandemic university landscape has made difficult to ignore, including socioeconomic pressures, digital constraints, and disruptions in social connectedness. In psychometric terms, the results support a multidimensional organization of stressors and a bifactor representation of stress reactions, with adequate internal consistency across subscales and evidence of temporal stability in the test-retest subsample. Considered together, these findings position BASE-66 as a broader, context-sensitive approach to AS assessment that aligns with contemporary higher education conditions, while maintaining conceptual continuity with process-based accounts of AS.

The EFA of the stressor pool supported a five-factor structure, reinforcing the multidimensional nature of AS in this sample and clarifying how distinct sources of demand cluster in contemporary university settings. The first factor, Academic Workload Stressors, maps onto the academic-demand core described in the SISCO-AS and SISCO-II-AS frameworks, but is operationalized here through items that better reflect the current organization of university work, including high evaluation load, limited time, and multiple simultaneous tasks. A second factor, Academic Performance Stressors, captured appraisals centered on understanding course content, meeting performance expectations, and the perceived risk of failing, which are key evaluative processes in process-based accounts of AS. Importantly, the emergence of three additional factors indicates that the stressor landscape relevant to AS cannot be reduced to academically anchored demands alone. Instead, the factorial structure expands the BASE-66 framework toward contextual pressures that have become particularly salient in the post-pandemic university environment, supporting the view that AS is shaped not only by academic requirements, but also by socioeconomic conditions, socio-technological transitions, and the quality of social interactions that organize students’ daily academic life. Recognizing this complexity is essential for understanding how different stressors converge and affect students’ current experiences, and it reinforces the need for more precise and context-sensitive assessments of AS [60–62].

Specifically, a Social Interaction Stressors factor captured strain linked to reduced social activity and longing for university life, indicating that disruptions in peer connectedness remain a meaningful source of pressure alongside academically driven demands. In parallel, Socioeconomic Stressors grouped items reflecting financial constraints and competing responsibilities, positioning material conditions and role overload as structurally relevant elements in the experience of AS rather than peripheral correlates. Finally, a Classroom Interaction Stressors factor highlighted perceived limitations in student–student and student–teacher interaction during academic activities, pointing to the pedagogical and relational climate of courses as an additional source of strain that is distinct from general social functioning. Overall, this structure supports approaching AS as a phenomenon shaped not only by academic demands, but also by students’ material circumstances and the quality of their social and instructional interactions, underscoring the value of context-sensitive assessment in current higher education environments [60–62].

Our primary objective was to develop a comprehensive instrument for the assessment of AS in university contexts, capable of integrating academic and non-academic sources of stress within a single measurement framework. In this effort, coping required particularly careful treatment. Prior work has documented limitations in the coping subscales of existing instruments, including the SISCO-AS and SISCO-II-AS, especially regarding factorial stability and conceptual coherence [17,19,59]. In the present study, coping indicators suggested a distinction between a restorative component oriented toward calming and recovery and a distraction–reappraisal component, yet the measurement properties of these dimensions suggest that coping remains less robust than the stressor and reaction pools. In substantive terms, this pattern is coherent with the view that coping strategies often operate as context-dependent responses whose expression varies with demands, timing, and available resources, rather than as stable latent dimensions. For this reason, coping results were interpreted conservatively, emphasizing their exploratory value and their potential for refinement, while the core psychometric conclusions of the BASE-66 focused on the stressor and reaction structures, which demonstrated substantially stronger structural consistency [17,19,59].

The emergence of Socioeconomic Stressors as a distinct factor underscores the central role of students’ material and contextual conditions in shaping AS experiences. This domain captures pressures linked to financial constraints, work obligations, and limited access to resources, which are not peripheral but structurally embedded in the academic trajectory of many university students. Prior research has shown that socioeconomic disadvantage is associated with higher levels of stress, poorer academic outcomes, and greater vulnerability to mental health difficulties [63]. In this sense, the explicit inclusion of socioeconomic stressors in BASE-66 constitutes a substantive advance over earlier instruments, which have tended to privilege academically anchored demands while positioning contextual constraints as secondary or external influences. This result reinforces a core implication of the model advanced here: AS should be approached as a process shaped by the interaction between academic requirements and the concrete conditions that organize students’ daily lives, including the availability of time, resources, and support.

The identification of a factor associated with Classroom Interaction Stressors further supports this broader reading. This domain demonstrated consistent loadings across evaluations, suggesting that its influence is tied to specific instructional contexts, pedagogical dynamics, and interactional climates that characterize particular courses and, in some cases, disciplinary cultures. Notably, no gender-based differences were observed for this factor, which may indicate a shared experience among male and female students in relation to classroom interaction demands, at least within this sample and context [64,65]. Considered together with the socioeconomic and social interaction domains, these findings emphasize that AS reflects not only academic overload or performance demands, but also from material pressures and relational dynamics that shape how students engage with university life. In that sense, BASE-66 is positioned to capture an expanded stress landscape that is more consistent with contemporary higher education conditions, while retaining interpretability at the domain level for applied use [63–65].

Beyond its conceptual relevance, this factor also has interpretive value because it clarifies why AS cannot be understood exclusively through workload and performance demands. Material constraints shape the conditions under which students study, connect, and participate in academic life, and they can amplify stress by narrowing the range of available coping resources and by increasing role overload. In this sense, the socioeconomic domain aligns with the broader aim of BASE-66 to capture the interaction between academic and non-academic strain, framing socioeconomic pressures not as background noise but as a structuring context that conditions both exposure to stressors and the likelihood that demands translate into reactions. These findings reinforce the importance of assessing AS as a phenomenon that is simultaneously academic and socio-contextual, and they support further investigation into how financial insecurity, employment demands, and caregiving responsibilities interact with academic stress processes, particularly in settings where inequalities remain a salient organizing feature of higher education [63].

In the analysis of stress reactions, a dominant general factor emerged that encapsulated manifestations commonly associated with both acute stress and burnout, mirroring the high intercorrelations among reaction subscales reported in prior work using SISCO-II-AS based instruments [17,19]. This general factor accounted for most of the observed variance, as reflected in the high explained common variance and hierarchical omega values obtained in the bifactor analysis, supporting the interpretation that stress reactions in university students tend to operate primarily as a generalized response rather than as sharply separated symptom clusters. At the same time, the delineation of three specific factors, emotional-cognitive reactions, somatic exhaustion, and social conflict, underscores the heterogeneous expression of AS, capturing cognitive-affective strain, fatigue-related physiological burden, and interpersonal or behavioral dysregulation as distinguishable profiles of response. Taken together, these results highlight the need to consider both the global burden of stress and its domain-specific expression when evaluating AS, and they suggest that BASE-66 can accommodate both parsimonious global assessment and more differentiated profiling, depending on whether the goal is screening, explanatory modeling, or the design of targeted educational and psychological support strategies [17,19].

The third factor captures stressors associated with general social interactions with friends and peers, highlighting interpersonal dynamics as a substantive source of AS. Although social relationships are often framed as protective, the present structure suggests that they may also function as persistent sources of strain, particularly under conditions of heightened academic demand. The social isolation imposed during the COVID-19 pandemic, together with the documented increase in loneliness among university students [66], likely contributed to the salience of this domain in the item pool and in participants’ responses. At the same time, the conceptual coherence of this factor supports the view that social interaction stressors reflect more enduring vulnerabilities in how students experience connectedness and social participation within university life, rather than being only a transient artifact of emergency conditions. In this sense, the presence of this factor strengthens the interpretation of BASE-66 as capturing an ecology of AS in which academically anchored demands coexist with relational pressures that shape both stress appraisal and access to supportive resources.

In parallel, the coping results allow a more precise distinction between restorative coping and distraction-reappraisal coping, which helps refine how coping is represented within the broader process of AS. Restorative coping, oriented toward calming and recovery, showed a negative association with emotional-cognitive reactions, suggesting that these strategies may specifically buffer maladaptive cognitive stress responses rather than overall stress intensity. In contrast, distraction-reappraisal coping was positively associated with physical exhaustion and the general reaction factor, indicating that these strategies may be mobilized under higher stress load and may coexist with fatigue rather than mitigate it. Nevertheless, the interpretation of the restorative factor is limited by item ambiguity, particularly for items referring to eating and substance use, which may reflect both adaptive regulation and maladaptive coping and therefore require further conceptual and psychometric refinement. Moreover, the distraction-reappraisal factor is underrepresented, and its low reliability indicates the need to expand this scale with additional items to better capture the construct. Importantly, these limitations in coping measurement mirror prior shortcomings identified in the coping subscales of existing instruments, including SISCO-AS and SISCO-II-AS, reinforcing the view that coping may behave as a more context-dependent and less structurally stable component within AS assessment [17,19].

However, the presence of this factor should not be interpreted only as a situational artifact of the pandemic. Its coherence within the stressor structure supports the view that vulnerability to social stress reflects more enduring features of students’ social functioning in university life, including the perceived availability of peer connectedness, the quality of everyday interactions, and the strain associated with reduced social participation. In this sense, the construct is better understood as part of the broader ecology of AS, where relational demands and disruptions can operate alongside academic pressures, shaping both the appraisal of stressors and the resources available to manage them [66].

Our study also revealed notable gender-based differences in response profiles, corroborating evidence suggesting that stress appraisal and stress-related responding may vary meaningfully by gender as a function of biological, psychological, and sociocultural influences [41–43,67]. Rather than implying a deterministic account, this pattern supports a biopsychosocial reading in which gendered role demands and differential appraisal processes shape how academic strain is perceived and expressed. From an applied standpoint, these findings underscore the relevance of incorporating a gender-sensitive perspective when evaluating AS, particularly in contexts where screening, prevention, and support strategies may need to be calibrated to differential stress profiles.

In parallel, the strong association between students’ self-perceived stress and overall stress reactions, together with its linkage to general stressor exposure, provides convergent evidence that subjective stress awareness is meaningfully embedded in broader AS processes [45]. This convergence suggests that brief self-perception indicators can serve as pragmatic complementary signals of distress in educational settings, especially when time or feasibility constraints limit the use of longer instruments. At the same time, the added value of BASE-66 lies in its capacity to move beyond global perception by identifying which stressor domains are most salient and which reaction profiles are most pronounced, enabling a more interpretable and actionable assessment of why a high perceived burden emerges and where intervention efforts may be more efficiently directed.

Taken together, these results represent an important step in the development and initial validation of BASE-66, an instrument designed to assess AS among Chilean university students through a broader and more context-sensitive framework that integrates academically anchored demands with socio-contextual pressures. The heterogeneity of the student sample, drawn from multiple universities and academic areas, supports the potential applicability of the inventory across diverse higher education settings, while also indicating that the identified domains are not confined to a single institutional or disciplinary context. Nevertheless, further studies are warranted to confirm generalizability in other student populations and to strengthen the evidence base through replication and subgroup-focused analyses.

The fourth factor, related to classroom interaction, showed a stable and well-defined structure, suggesting that this stressors are embedded in specific academic settings rather than in broader social contexts. These stressors likely involve pedagogical dynamics, communication practices, and the interactional climate that emerges within particular courses and, in some cases, within disciplinary cultures. In practical terms, this domain points to stressors that are not only about what students must do, but also about how academic participation is organized and mediated, including the perceived responsiveness of instructors and the extent to which interaction is facilitated or constrained during academic activities.

No gender differences were observed for this factor. This pattern suggests that classroom interaction stressors may be experienced in a relatively similar manner by female and male students, at least within this sample and context. Rather than implying that gender is irrelevant to classroom life, the absence of differences may indicate that these stressors are anchored in shared structural features of instructional contexts, such as interaction opportunities, participation norms, and communication channels, which affect students broadly. This finding aligns with prior work reporting limited or non-systematic gender differences in some academic-contextual stress domains [64,65].

The identification of a distinct classroom interaction factor reinforces the view that AS is not generated exclusively by workload or performance demands, but also by the quality of relational and communicative processes that shape everyday academic engagement. This distinction is conceptually important because it separates general social strain from interaction stressors that are specifically tied to the instructional environment, where students’ learners roles, the authority structure, and evaluation-related dynamics converge. Accordingly, BASE-66 captures a broader and more differentiated stress landscape in which academic, social, and contextual dimensions jointly contribute to students’ AS experiences, and where classroom interaction can be recognized as a meaningful and potentially modifiable source of strain [64,65].

In the analysis of stress reactions, a dominant general factor emerged, encompassing manifestations commonly associated with both acute stress and burnout. This pattern mirrors the high intercorrelations among reaction subscales reported in previous studies using SISCO-II-AS-based instruments [17,19,59]. The predominance of this general factor was supported by bifactors indices. High explained common variance and a strong hierarchical omega for the general dimension suggest that a substantial proportion of the variability in reactions is shared across symptom domains, which supports interpreting reactions primarily as a generalized response to AS rather than as sharply segmented clusters.

At the same time, the bifactor solution retained specific domains with interpretable content and non-trivial reliable variance after controlling for the general dimension. In substantive terms, the Emotional-Cognitive Reactions domain captures cognitive-affective strain expressed through intrusive thoughts, worry, and dysregulated emotional responding in relation to AS demands. The Physical Exhaustion Reactions domain reflects a somatic and fatigue-related profile, consistent with the physical cost of sustained academic requirements and cumulative overload. Finally, Social Conflict Reactions capture an interpersonal and behavioral expression of strain that may become visible in irritability, tension, and conflict in social functioning. This configuration is coherent with the idea that, even when a broad stress reaction factor dominates, students may differ in how that burden is expressed, either through more cognitive-emotional disruption, more somatic-exhaustive manifestations, or more socially dysregulated responses.

From an applied and interpretive standpoint, this structure supports using the general reaction score as a parsimonious indicator of overall stress burden, particularly when screening or global monitoring is the priority. However, the presence of distinct subdomains also justifies retaining specific scores when the aim is to characterize profiles, tailor support strategies, or evaluate targeted interventions. In this sense, BASE-66 can operate at multiple levels of analysis, offering both a global index of overall reaction intensity and complementary information about the predominant mode of response, which may be especially relevant in educational and psychological settings where the functional consequences of stress vary across students [17,19,59].

Convergent validity was supported by the theoretically coherent alignment between stressor exposure and stress reactions. At the general score level, the association between Total Stressors and the general reactions factor is conceptually expected, because both indices represent linked stages of the same process: the appraisal of demands as stressors and the systemic imbalance expressed through reactions [14]. In this sense, convergence between overall stressor burden and overall reaction intensity should not be interpreted as redundancy, but as a process-consistent coupling between construct-relevant components and an expected form of shared variance in AS assessment [54].

At the same time, discriminant validity is best evaluated at the domain level, where BASE-66 preserves meaningful differentiation across sources of strain and reaction profiles. The separation among stressor subscales supports the interpretation that academically anchored demands, socioeconomic constraints, and social or instructional interaction pressures are related but not interchangeable facets of the AS experience. Similarly, although the general reactions factor dominates the reaction pool, the presence of interpretable specific domains supports a differentiated interpretation of how stress is expressed beyond a single severity continuum [54].

This configuration has direct interpretive implications. When screening or global monitoring is the goal, aggregated scores provide a parsimonious estimate of overall AS burden, and this shared variance is informative rather than problematic. However, when the goal is explanatory modeling or intervention design, domain-specific scores add value by indicating which stressor sources are most salient and which reaction profiles are most pronounced. In practical terms, BASE-66 supports a two-level approach, combining global indices that summarize the overall load of the AS process with specific scores that retain sufficient differentiation to guide targeted interpretation and support strategies [14,54].

Building on this two-level interpretive framework, we also observed systematic group differences that underscore the practical relevance of domain-level scores. Specifically, women reported higher levels across several AS domains, aligning with prior evidence that stress appraisal and stress-related responding may differ by gender as a function of biological, psychological, and sociocultural influences [41–43,67]. In this sample, women reported higher levels on key academic stressor dimensions and on the general reaction score, with differences also extending to specific reaction profiles. This pattern is consistent with a biopsychosocial reading in which gendered exposure, role demands, and differential appraisal processes shape how academic strain is perceived and expressed, rather than implying a deterministic or exclusively biological mechanism. From a measurement perspective, these findings reinforce the relevance of adopting a gender-sensitive approach in AS assessment, particularly in applied settings where screening and support strategies may need to be calibrated to differential stress profiles.

In parallel, the association between the single-item measure of self-perceived nervousness and both overall stress reactions and stressor exposure provides additional convergent evidence that subjective stress awareness is meaningfully linked to broader AS processes [45]. These correlations suggest that this brief indicator is not merely a generic affective signal, but rather co-varies in a theoretically coherent way with both the intensity of stressors and the magnitude of reactions. From an applied standpoint, this convergence supports the pragmatic value of short self-perception indicators as complementary signals of distress in educational contexts, particularly when time constraints limit the feasibility of longer instruments. At the same time, the added value of BASE-66 lies in its capacity to move beyond global perception by identifying which domains are most salient for each student, which is important for interpreting why a high perceived burden emerges and where intervention efforts may be more efficiently directed.

Taken together, these findings support conceptualizing AS as a multidimensional phenomenon shaped by individual and contextual factors. They also reinforce the value of assessment tools that can capture this complexity in a structured and interpretable way, combining global indicators of distress with domain-specific information that can inform targeted prevention and support efforts [41–43,67].

This study represents an key step in the development and initial validation of BASE-66 as a measure designed to assess AS in Chilean university students. By expanding content coverage beyond traditionally academic demands, the instrument addresses gaps in existing approaches and supports a broader, context-sensitive assessment of stress experiences that is better aligned with contemporary higher education conditions. The heterogeneity of the sample, drawn from multiple universities and academic areas, strengthens the plausibility of its use across diverse institutional settings, while also indicating that the identified domains capture stressors and reactions that are not confined to a single disciplinary culture.

At the same time, this initial validation should be interpreted as a foundation rather than an endpoint. Additional studies are needed to evaluate measurement invariance across key subgroups and to examine generalizability in other student populations and institutional contexts, including replication in independent samples. Establishing invariance is particularly relevant given the gender differences observed in several domains and the contextual nature of multiple stressor dimensions, because it would clarify whether observed group differences reflect substantive variation in AS processes rather than measurement artifacts.

However, several limitations should be considered when interpreting these findings. Although BASE-66 broadens the assessment of AS, it may not capture the full range of stressors and reactions experienced by all student groups across institutional contexts. In addition, the evidence is based primarily on a single measurement occasion for the full sample, which limits inferences about the temporal dynamics of stress responses. While the test–retest analyses provided initial support for score stability in a subsample, these results should be interpreted cautiously given reduced follow-up participation and the likelihood that some domains are context-sensitive.

Finally, reliance on self-report may introduce shared-method variance and reporting biases. Future work should incorporate complementary assessment strategies, including objective stress indicators and physiological measures, to strengthen construct validation and clarify links between perceived stress and biological stress-related processes [6]. More rigorous longitudinal designs and formal tests of measurement invariance will also be necessary to confirm the utility of BASE-66 across diverse educational contexts.

In summary, this study contributes to educational psychology and student mental health by introducing BASE-66 and providing initial psychometric evidence for an instrument designed to support a comprehensive assessment of AS by integrating academic and non-academic sources of strain within a single framework. The findings reinforce that AS is best understood as a multidimensional phenomenon in which academically anchored demands coexist with contextual pressures tied to students’ material conditions and to social and instructional interactions. The observed gender differences further underscore the relevance of adopting a gender-sensitive perspective when interpreting stress exposure and stress-related responding in university settings. Together, these results provide a foundation for future research to confirm the robustness and generalizability of BASE-66 across settings and over time, while highlighting its potential utility for more targeted screening and intervention efforts addressing distinct stress profiles in higher education.

## Supporting information

S1 Table Stressors (BASE-66): factor loadings and domain structure. Items (Str) and their descriptions with standardized loadings across the five stressor domains: Academic Workload Stressors, Academic Performance Stressors, Social Interaction Stressors, Socioeconomic Stressors, and Classroom Interaction Stressors.
(XLSX)

S2 Table Reactions (BASE-66): bifactor model loadings. Items (Rea) and their descriptions with standardized loadings for the general factor and three specific factors: Emotional-Cognitive Reactions, Physical Exhaustion Reactions, and Social Conflict Reactions.
(XLSX)

S3 Table Coping (BASE-66): factor loadings and coping domains. Items (Cop) and their descriptions with standardized loadings across two coping domains: Restorative coping, and Distraction and reappraisal coping.
(XLSX)

S4 File Focus group-derived categories supporting item development. Categories resulting from the focus group analysis used during content development of the instrument, organized into Stressors, Reactions, and Coping domains.
(PDF)

S1 Appendix BASE-66 inventory (English version). Complete instrument for research use.
(PDF)
