# Supplementary material for: Development and validation of the BASE-66 inventory for comprehensive academic stress measurement
Source: PLoS One. 2026 Mar 11;21(3):e0343308. doi: 10.1371/journal.pone.0343308 (PMC12978457; doi:10.1371/journal.pone.0343308)
Supplement: S4 File — Categories resulting from the focus group analysis used during content development of the instrument, organized into Stressors, Reactions, and Coping domains. (PDF) [file pone.0343308.s004.pdf]

Categories resulting from the focus group analysis.

Stressors

Nº	Category	Description
1	Academic career management	Problems in career management, such as coordinating activities, communications, etc.
2	Isolation	Poor social interaction with friends and/or peers
3	Learning	Negative learning and/or performance expectations or results
4	Connectivity	Problems with internet connectivity
5	Coordination with peers	Problems coordinating with peers in academic activities
6	Distraction	Presence of distractions at home, such as household noise, interruptions from family members, presence of pets, etc.
7	Characteristics of teacher pedagogy	Teaching methodologies, feedback and evaluation from teachers.
8	Lack of environmental support	Lack of support from my family and/or friends.
9	Lack of time with family and/or friends	Lack of time to spend with family and/or friends.
10	Missing university	Missing university due to lack of contact with peers and lack of access to university resources.
11	Institutional management	Problems in the management of the university institution, such as coordinating activities, communications, etc.
12	Domestic obligations	Compatibility of domestic and academic work.
13	University platforms	Complexity, stability and general functioning of the university's e-learning platforms.
14	Privacy	Exposure in front of others in class
15	Financial problems	Lack of financial resources to pay for the course and/or internet connectivity.
16	Lack of interaction in classes	Problems of interaction between students and teacher, as well as from teacher to students (teacher asks questions and nobody answers).
17	Lack of physical resources	Lack of physical space to study
18	Health problems	Presence of physical and mental health problems
19	Screen time	Too much time in front of a screen

Reactions

Nº	Category	Description
1	Exhaustion	Physical and mental exhaustion
2	Alopecia	Hair loss and/or hair loss
3	Feelings of guilt (for not studying):	Guilt about not studying and/or not performing well enough
4	Cognitive problems	Problems with attention, concentration, and memory
5	Digestive problems	Problems with digestion, abdominal pain, nausea or diarrhoea
6	Feelings of worthlessness	Feelings of inadequacy and/or worthlessness with regard to studies
7	Visual symptoms	Eye problems (eye strain, blurred vision, dryness, palpitations, irritation)
8	Tics	Involuntary body movements, tics (involuntary movement of legs, throbbing in eyes or faces)
9	Changes in sexuality	Increased or decreased sexual desire
10	Sweating	Excessive sweating
11	Tremors	Tremors in part or all of the body
12	Tendonitis	Pain in the tendons of the hands, mainly from keyboarding
13	Warmth in the ears	Feeling of heat in the ears
14	Vertigo	Feeling of vertigo
15	Tinnitus	Recurrent or permanent ringing in the ear
16	Dry mouth	Dryness of the mouth
17	Breathing disturbances	Feeling of shortness of breath, suffocation

Coping

Nº	Category	Description
1	Self-praise and awards	The person gives awards or praise to him/herself.
2	Seeking professional support	Seeking professional support to cope with stress
3	Use of food, drink and other substances	Use of different types of food, drink and other substances to calm down or stimulate oneself
4	Self-pity	Being empathetic and compassionate with oneself, trying to forgive oneself
5	Resting	Sleeping and/or resting before or after a stressful task
6	Distracting oneself with work	Working harder, so as not to perceive stress
7	Religious	Ask for help from God, pray and/or attend religious masses or services; ask for spiritual support
8	Procrastination	Avoiding or postponing academic tasks, doing other things
9	Physical space management	Organise my work space and resources
10	Peace of mind	Seek tranquillity / a quiet space
