# Supplementary material for: Development and validation of the BASE-66 inventory for comprehensive academic stress measurement
Source: PLoS One. 2026 Mar 11;21(3):e0343308. doi: 10.1371/journal.pone.0343308 (PMC12978457; doi:10.1371/journal.pone.0343308)
Supplement: S1 Appendix — Complete instrument for research use. (PDF) [file pone.0343308.s005.pdf]

Broad Academic Stress Evaluation BASE-66

This questionnaire aims to recognize the characteristics of stress in college students, combining academic and non-academic factors for a complete and accurate assessment of their overall well-being and mental health.

1. With the idea of obtaining greater accuracy and using a scale from 1 to 5, indicate your level of worry or nervousness, where (1) is a little and (5) a lot.

1	2	3	4	5

2. During the course of this semester, have you had moments of worry or nervousness?

Yes ☐ No ☐

3. On a scale from (1) to (5), where (1) is never, (2) is rarely, (3) is sometimes, (4) is almost always, and (5) is always, indicate how often the following situations made you anxious:

Stressors					
	(1)	(2)	(3)	(4)	(5)
	Never	Rarely	Some times	Almost always	Always
**Academic Workload Stressors**					
Overload of homework and academic work					
Lack of time to spend with family and/or friends due to the academic burden.					
Teachers' evaluations (exams, essays, research papers, etc.)					
The type of work teachers ask you to do (map consultation, worksheets, essays, concept maps, etc.).					
Limited time to do the work					
**Academic Performance Stressors**					
Not understanding the topics covered in class					
Group mates make faster progress on assignments and/or academic work.					
Not being satisfied and/or not meeting my expectations with my academic performance.					
Possibility of failing one or more subjects					
Class participation (answering questions, presentations, etc.).					
**Social interaction Stressors**					
Reduced social interactions with friends and/or classmates					
Low participation in social activities					

Little university social life					
Missing or longing for university life					
**Socioeconomic Stressors**					
Having to work to pay for my studies and/or household expenses.					
Lack of resources to pay for internet connectivity.					
Lack of resources to renew equipment for academic work.					
Balancing other responsibilities (domestic and/or work) with my academic activities.					
Lack of help with health, social and/or economic problems.					
Lack of adequate physical space to study					
Lack of support from my family and friends with my academic duties					
**Classroom Interaction Stressors**					
Lack of interaction between students and teachers in class					
Lack of response to questions and/or activities proposed by the teacher.					
Lack of interaction between students in class					

4. On a scale from (1) to (5), where (1) is never, (2) is rarely, (3) is sometimes, (4) i is almost always, and (5) is always, indicate how often you experienced each reaction when facing academic stress.

**Reactions**					
	(1)	(2)	(3)	(4)	(5)
	**Never**	**Rarely**	**Some times**	**Almost always**	**Always**
Feelings of depression and sadness (low mood)					
Physical and mental exhaustion					
Anxiety, distress or despair					
Chronic fatigue (permanent tiredness)					
Restlessness (inability to relax and be calm)					
Unwillingness to do your work as a student					
Sleep disorders (insomnia or nightmares)					
Breathing disturbances (shortness of breath, suffocation).					
Increased or decreased food intake					
Isolation from others					
Drowsiness or increased need for sleep.					
Headaches or migraines					
Digestion problems, abdominal pain or diarrhoea					
Skin reactions (rash, peeling, etc.)					
Scratching, nail biting, rubbing, etc.					
Eye problems (eye strain, blurred vision, dryness, palpitations, irritation)					
Increased or decreased sexual desire					
Hair loss					
**Emotional-Cognitive Reactions**					
Feelings of inadequacy and/or uselessness with regard to studies					
Problems with attention, concentration and/or memory					
Feelings of guilt for not fulfilling my academic activities according to my expectations					
**Physical Exhaustion Reactions**					
Tremors					
Warmth in the ears					
Vertigo					
Sweating					
Involuntary body movements, tics (involuntary movement of legs, throbbing in eyes or faces)					
Tendinitis					
Tinnitus (recurrent or permanent ringing in the ear)					
Dry mouth					
**Social Conflict Reactions**					
Conflict or tendency to argue or dispute					
Feelings of aggression or increased irritability					
Sudden mood swings					

5. On a scale from (1) to (5), where (1) is never, (2) is rarely, (3) is sometimes, (4) i is almost always, and (5) is always, indicate how often you used each of the following strategies when you felt academically stressed.

Reactions					
	(1)	(2)	(3)	(4)	(5)
	Never	Rarely	Some times	Almost always	Always
**Restorative coping**					
Sleep and/or rest before or after a stressful task					
Consume healthy food and/or beverages to energize and/or calm myself.					
Use substances (alcohol, tobacco, drugs, medications) to energize and/or calm myself.					
Seek calm by finding a quiet space.					
Be empathic and compassionate toward myself; try to forgive myself.					
**Distraction and reappraisal coping**					
Engage in a pastime (physical activity, reading, watching series, social media, etc.).					
Consume junk food and/or beverages to energize and/or calm myself.					
Try to find something positive or beneficial in the stressful situation.					
